# Dynamic Clustering of the Bacterial Sensory Kinase BaeS

**DOI:** 10.1371/journal.pone.0150349

**Published:** 2016-03-07

**Authors:** Moriah Koler, Vered Frank, Hadar Amartely, Assaf Friedler, Ady Vaknin

**Affiliations:** 1 The Racah Institute of Physics, The Hebrew University of Jerusalem, Safra Campus, Givat Ram, Jerusalem, 91904, Israel; 2 The Institute of Chemistry, The Hebrew University of Jerusalem, Safra Campus, Givat Ram, Jerusalem, 91904, Israel; University of Illinois at Urbana-Champaign, UNITED STATES

## Abstract

Several bacterial sensory-kinase receptors form clusters on the cell membrane. However, the dynamics of sensory-kinase clustering are largely unclear. Using measurements of fluorescence anisotropy and time-lapse imaging of *Escherichia coli* cells, we demonstrate that copper ions trigger self-association of BaeS receptors and lead to rapid formation of clusters, which can be reversibly dispersed by a metal chelator. Copper ions did not trigger self-association of other fluorescently tagged sensory kinases, and other divalent metal ions could not elicit self-association of BaeS. The histidine residues in the BaeS periplasmic domain are essential for copper binding *in vitro* and are important for the copper-induced BaeS responses *in vivo*. BaeS clustering was triggered also under conditions that directly triggered BaeS-dependent transcriptional responses. Thus, clustering of sensory kinase receptors can be dynamic and context dependent and can be triggered by specific environmental cues.

## Introduction

Transmembrane sensory signaling in bacteria is often mediated by histidine-kinase receptors. These receptors generally have an external sensor domain and internal cytoplasmic domains that function as a kinase, and in some cases also as a phosphatase. Environmental cues ultimately affect the phosphate transfer between the sensor and a response regulator protein, which, in most cases, acts as a transcription factor. A single bacterium can contain as many as several hundred types of such sensory kinase/response-regulator pairs, also known as two-component sensory systems. These sensory systems are involved in various functions, including stress responses, motility, metabolic responses, quorum sensing, development, drug resistance and pathogenicity [1–5]. In particular, the BaeS-BaeR system plays a role in the expression of the MdtABC and AcrD efflux pumps, as well as the periplasmic Spy protein, in response to various envelope stresses [6–14] and, in particular, in the response to copper [11], zinc [9, 13], and sodium tungstate [9]. Although the basic phosphoryl transfer scheme utilized by two-component sensory systems is generally well understood [15], other aspects of their function, including their spatial organization, are generally poorly understood [16].

Recent experiments in *E*. *coli* have revealed that a stimulus induces slow changes in the packing of chemoreceptors within clusters, leading to functional plasticity [17]. A more complex behavior has been observed in the chemotaxis system of *Bacillus subtilis*, where stimulus enhances the lateral clusters but weakens the polar clusters [18]. However, chemotaxis receptors are unique in that they interact with distinct cytoplasmic enzymes to mediate the kinase and phosphatase functions. During progression through the cell cycle, the CckA sensory kinase of *Caulobacter crescentus* forms clusters that play an essential role in the asymmetrical division of this bacterium [19]. Similarly, clusters of the YycG sensor from *B*. *subtilis* are associated with the division machinery [20]. More generic sensory kinases have also been reported to form clusters, including the DcuS, CitA, TorS, and EvgS sensors in *E*. *coli* and the RpfC sensor in *Xanthomonas campestris* [21–23].

Here, we demonstrate that clustering of the BaeS sensory kinase in *E*. *coli* is dynamic and controlled by specific external cues. We show that the BaeS sensors rapidly form clusters in the presence of low concentrations of external copper ions and reversibly disperse upon the addition of a metal chelator. Mutational analysis and *in vitro* binding studies suggested that clustering is triggered by direct interaction of copper ions with four histidine residues in the BaeS periplasmic domain. BaeS clustering was clearly triggered by other conditions that promoted also BaeS-mediated transcriptional responses, including in the presence of sodium tungstate at lower pH or in combination with copper. These findings indicate that clustering of sensory histidine-kinases in bacteria can be dynamic and responsive to environmental cues.

## Results

### Testing the mYFP-tagged BaeS sensors

**Fig 1:**
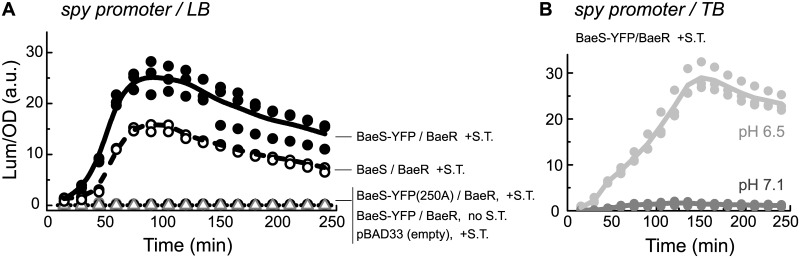
The mYFP-tagged BaeS sensors mediate transcriptional responses of the *spy* promoter to sodium tungstate. Time traces of luminescence, normalized to the optical density (OD_600_) of the culture, measured from Δ*baeS* cells (JW2063C) carrying the *lux* system under control of the *spy* promoter before and after the addition, at t = 0, of 10 mM sodium tungstate. Cells contained either an empty pBAD33 plasmid, a plasmid encoding the tagged sensor BaeS-mYFP, a plasmid encoding the mutant sensor BaeS^H250A^-mYFP, or a plasmid encoding the untagged BaeS sensor. In all cases, the *baeR* response regulator gene was also cloned in tandem. Measurements were performed in LB medium (**A**) or TB medium (**B**). Each experiment was repeated three times.

To monitor the self-association of the BaeS sensory kinase, we tagged it with monomeric yellow fluorescent protein EYFP^A206K^ (mYFP) at its C-terminal cytoplasmic end. This monomeric variant of YFP has an immeasurably low tendency for self-association [24]. To test the capacity of the mYFP-tagged BaeS sensors to promote transcriptional responses, we used a Δ*baeS* (JW2063C) strain supplemented with a pBR2TTS plasmid carrying the *lux* system under the control of the *spy* or *acrD* promoter and measured the respective luminescent responses to sodium tungstate (Fig 1) or indole (S1 Fig) [8, 9, 13]. Experiments were performed in the commonly used LB medium. When these cells were supplemented with an empty pBAD33 plasmid, no response was observed with either promoter. However, when *baeS-mYFP* and *baeR* were cloned in tandem into this plasmid we could measure a clear response of the *spy* promoter to sodium tungstate and of the *acrD* promoter to indole. The *baeR* gene was required, possibly because the expression of BaeR under the conditions of the experiment is too low or in order to maintain a proper ratio between BaeS and BaeR. These transcriptional responses clearly relied on the presence of sodium tungstate or indole, indicating that the enhanced promoter activity is not promoted solely by the addition of the response regulator *baeR*. In addition, a single replacement (H250A) at the phosphorylation site of BaeS eliminated the response of the *spy* promoter and led to a substantial reduction in the transcriptional responses of the *acrD* promoter. Thus, the tagged BaeS sensors could mediate the expected responses to both indole and sodium tungstate, indicating that the tagged sensor maintains its basic function.

We further tested the responses of the *spy* promoter to sodium tungstate in the less-rich medium Bacto tryptone (TB; BD Biosciences). When cells were grown in the TB medium, the response of the *spy* promoter to sodium tungstate was diminished (Fig 1B). However, we noted that the growth of the *E*. *coli* cultures in LB but not in TB leads to a reduction in the pH of the medium to approximately 6.5. Thus, we tested whether lower pH affects the response of the *spy* promoter to sodium tungstate. As shown in Fig 1B, reducing the pH of the TB medium to 6.5 restored the response of the *spy* promoter to sodium tungstate, suggesting that the transcriptional response of the *spy* promoter to sodium tungstate in LB relies on a moderately acidic environment.

### Copper-induced clustering of the mYFP-tagged BaeS sensors

**Fig 2:**
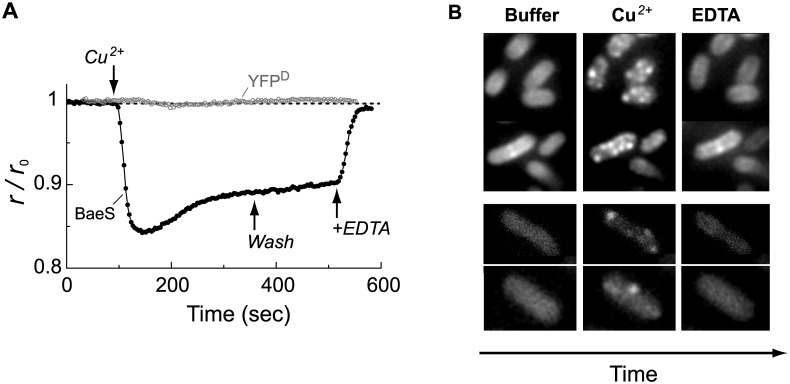
Copper-induced self-association of the BaeS sensory kinase. (**A**) Fluorescence anisotropy traces were obtained with Δ*baeS* cells expressing either mYFP-tagged BaeS (filled black symbols) or free mYFP dimers (YFP^D^; open gray symbols), induced with 5 μM IPTG. Arrows indicate the times at which CuSO_4_ (30 μM) was added or washed out and the times at which EDTA (100 μM) was added. (**B**) *Upper panel*: time-lapse images of similar cells as in (A) placed in a flow chamber and imaged successively, with approximately 300 sec delay between images, in buffer, buffer containing 30 μM CuSO_4_, and buffer containing 100 μM EDTA. *Lower panel*: similar time-lapse experiments performed with wild-type (MG1655) cells expressing the tagged BaeS sensors from a pBAD33 plasmid, using 0.001% arabinose. The expression level in the specific cells imaged here was approximately 100–200 sensors/cell (see Materials and Methods).

Self-association of the mYFP-tagged BaeS sensors was detected by monitoring changes in the anisotropy of the emitted fluorescence, which is sensitive to homo-FRET between the mYFPs [25, 26]. In addition, changes in the global clustering pattern of these sensors were monitored using time-lapse microscopy. Experiments were performed in a standard motility medium at pH 7 [27], with the EDTA metal chelator generally omitted, except when specifically mentioned. A typical anisotropy trace measured from mYFP-tagged BaeS sensors expressed in the Δ*baeS* strain is shown in Fig 2A (filled black symbols). The addition of CuSO_4_ (30 μM) led to a rapid and considerable decrease in the measured anisotropy, which corresponds to an increase in homo-FRET, thus indicating that these sensors became clustered in the presence of copper. Some slow recovery of anisotropy occurred in the presence of copper, and washing out the copper did not increase much the rate of recovery. However, the addition of EDTA (100 μM; upward arrow) led to an abrupt and complete recovery to the prestimulation level of anisotropy. EDTA, a known chelator of divalent metal ions, must efficiently compete for binding to Cu^2+^ ions. The response to CuSO_4_ (30 μM) was also measured with cells expressing a dimeric mYFP-mYFP (mYFP^D^) fusion protein in the cytoplasm (Fig 2A, open gray symbols). This is the most basic control that mimics the dimeric nature of the sensory kinases and the general location of the mYFP tag in the cytoplasmic space. Cu^2+^ had no effect on the anisotropy measured from mYFP^D^ in the cytoplasm, indicating that the BaeS sensor itself mediates the observed response to copper.

Given the considerable effect of copper on the BaeS sensors indicated by the fluorescence anisotropy measurements, we further explored the effect of copper ions on the global spatial distribution of these sensors using time-lapse fluorescence microscopy (Fig 2B). In the absence of a stimulus, the tagged BaeS sensors were distributed homogeneously over the cell membrane. Consistent with the fluorescence anisotropy data, the addition of Cu^2+^ (30 μM) led to the formation of distinct clusters of BaeS-mYFP. Moreover, the addition of EDTA led to the dissociation of the clusters and recovery of the pre-stimulus pattern of homogeneous distribution of the sensor. Similar responses were observed when the tagged BaeS sensor was expressed at much lower levels (100–200 sensors per cell) in the wild-type MG1655 cells in the presence of a low concentration of the inducer arabinose. The expression level of the tagged sensors was quantified as described by Sommer et al. [23] (Fig 2B, lower panel). Thus, copper ions and EDTA promoted the clustering and dispersal of the BaeS sensors, respectively.

### The dose-dependence of the BaeS response to copper ions

**Fig 3:**
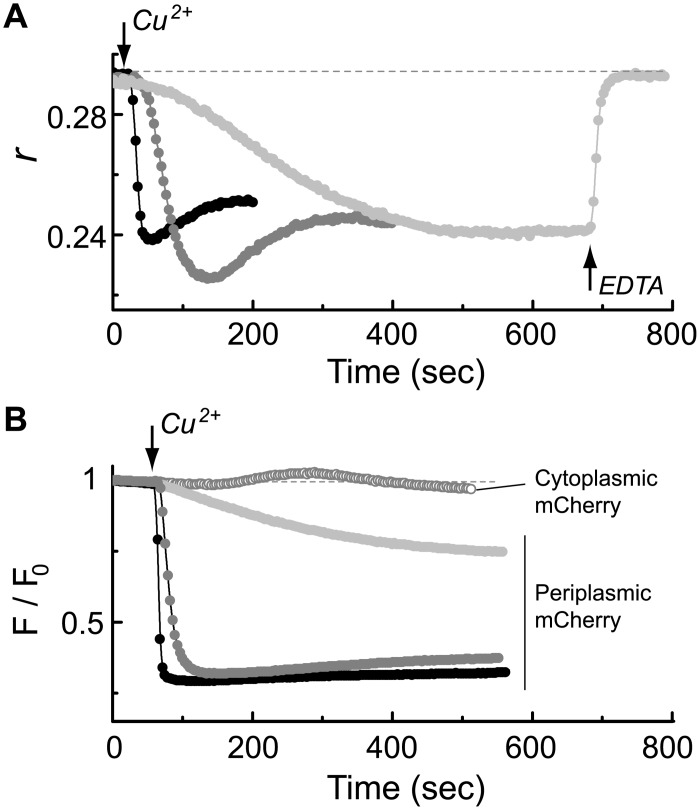
Dependence of the BaeS clustering on Cu^2+^ concentration. (**A**) Fluorescence anisotropy traces measured from Δ*baeS* cells expressing mYFP-tagged BaeS and challenged with increasing concentrations of CuSO_4_: 0.5 μM (light gray symbols), 10 μM (gray symbols), and 100 μM (black symbols). Copper was added at the time indicated by the arrow. For comparison, panel (**B**) presents the corresponding dynamics of the copper-induced quenching of mCherry in the cytoplasmic space (open symbols) or periplasmic space (filled symbols). The concentrations of CuSO_4_ are coded as in (A). mCherry was targeted to the periplasmic space by fusing it to the TorT periplasmic binding protein [23].

The dose-dependent response of the BaeS sensors to copper is shown in Fig 3A. The BaeS sensors responded to copper at concentrations below 1 μM. Increasing the added copper concentration mainly affected the dynamics of the response, which was considerably more rapid at higher concentrations.

In principle, copper ions can affect the BaeS sensors by its presence in either the periplasmic or the cytoplasmic space. Copper ions enter the periplasmic space through porins in the outer membrane of the bacterium. However, the mechanism by which copper enters the cytoplasmic space is not known [28]. Therefore, we sought to measure the dynamics of copper binding to a generic protein in either the periplasmic or cytoplasmic space to compare the results to the dynamics of the BaeS response. Based on the observation that the binding of copper ions to the red fluorescent protein HcRed effectively quenches its fluorescence [29], we constructed two strains expressing the red fluorescent protein mCherry: in one, mCherry freely diffused through the cytoplasmic space. In the other, mCherry was fused to a periplasmic binding protein (TorT) and was thus translocated to the periplasmic space [23]. The quenching dynamics of mCherry in these strains upon the addition of copper are shown in Fig 3B. The addition of Cu^2+^ (10 μM) led to a substantial and rapid quenching of the periplasmic mCherry (filled gray symbols) but only had a minor effect on the cytoplasmic mCherry (open gray symbols). In general, the quenching dynamics of the periplasmic mCherry appeared qualitatively similar to the fluorescence polarization response of BaeS (Fig 3A). These results, combined with the rapid recovery of anisotropy upon the addition of EDTA, support the suggestion that Cu^2+^ ions exert their effect on the BaeS sensor by binding to its periplasmic domain.

### Specificity of the copper-induced BaeS clustering

**Fig 4:**
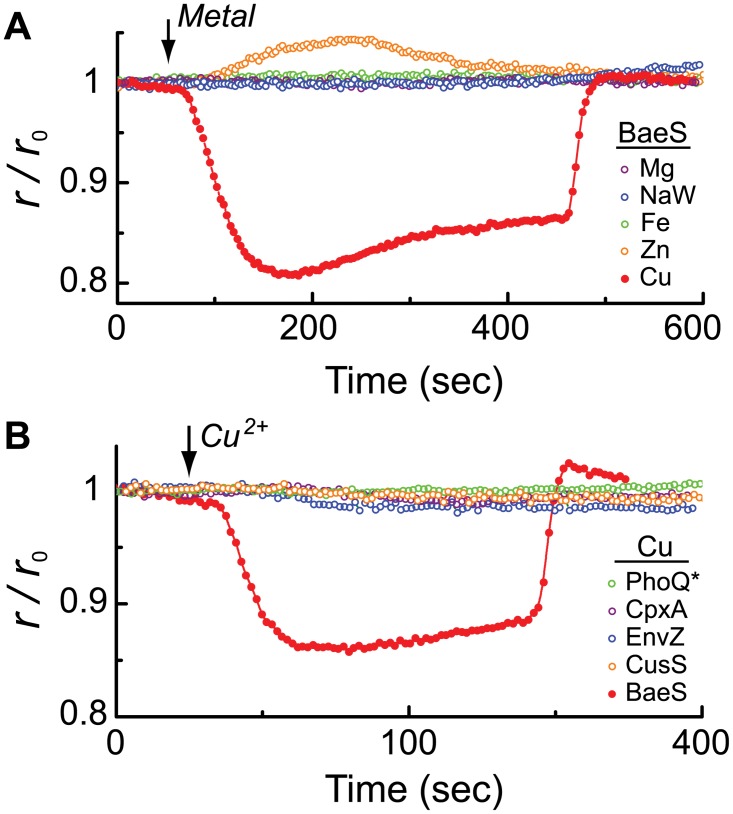
The specificity of the BaeS response to copper. (**A**) Fluorescence anisotropy traces measured from Δ*baeS* cells expressing mYFP-tagged BaeS. MgCl_2_, Na_2_WO_4_, FeSO_4_, or ZnSO_4_ (10 μM) was added at the time indicated by the arrow and removed 200 seconds later. The response to 10 μM CuSO_4_ is shown for comparison. (**B**) Anisotropy responses of different mYFP-tagged sensory kinases to CuSO_4_ (10 μM). To avoid possible differences among deletion strains, all of the tagged sensors were tested in wild-type strain MG1655, in which all sensors are naturally expressed from the chromosome.

To test whether the anisotropy response of BaeS to copper is specific or represents a common response of transmembrane sensors to metal ions, we performed two sets of experiments. In the first set, the BaeS sensors were challenged with other divalent metal ions, some of which were previously reported to affect BaeSR signaling (Fig 4A); in the second set, other sensory kinases from *E*. *coli* were challenged with copper (Fig 4B). As shown in Fig 4A, BaeS exhibited no response to Mg^2+^, Fe^2+^, or sodium tungstate, and only a weak response to Zn^2+^. The weak response to zinc was consistently opposite to the response triggered by copper. We then tested the response to copper of other mYFP-tagged sensory kinases. The CusS sensory kinase is known to play a role in copper sensing [28]. We also tested the CpxA sensor, which is involved in the envelope stress response [12, 30, 31], and the EnvZ sensor, which responds to osmotic stress [32]. Finally, we tested the PhoQ^N202R^ sensor, which is reported to be inactive [33] as a non-responsive control. All these sensors were compared in the same wild-type MG1655 strain to ensure that the observed effects were not caused by the use of different deletion strains. As shown in Fig 4B, none of these sensory kinases responded to copper like the BaeS sensor. However, a small anisotropy response and a corresponding weak clustering tendency were observed when a higher concentration of Cu^2+^ (100 μM) was used to stimulate the copper-sensing CusS sensor (S2 Fig). Thus, the sensitivity of BaeS to copper clearly relies on specific properties of this sensor.

### The role of the periplasmic sensor domain of BaeS in its response to copper

The dynamics of the BaeS response to copper and the rapid recovery upon the addition of EDTA (Figs 2 and 3) suggested that copper confers its effect in the periplasmic space. Histidine residues have been reported to play a role in the copper-induced formation of amyloid fibers [34, 35] and in the binding of copper to the periplasmic sensor domain of the CinS sensory kinase in the bacterium *Pseudomonas putida* [36]. Thus, we tested whether copper ions interact directly with the periplasmic sensor domain of BaeS and whether histidine residues play a role in the observed response.

**Fig 5:**
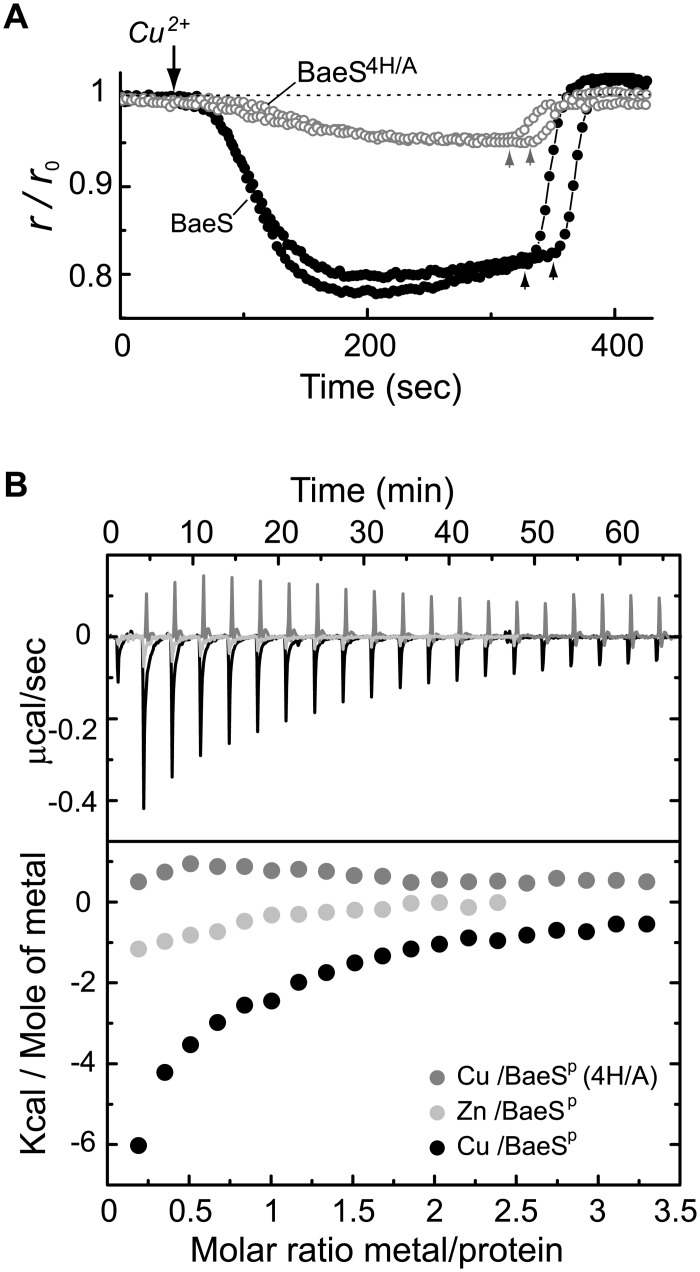
The effect of His/Ala replacement in the periplasmic domain of BaeS. (**A**) Fluorescence anisotropy traces measured from Δ*baeS* cells expressing tagged BaeS sensors (black symbols) or mutant BaeS^H4/A^ sensors (gray symbols). The BaeS^H4/A^ mutant contains four His-to-Ala replacements in its periplasmic domain, at positions 45, 65, 87, and 101. Copper (10 μM) and EDTA (100 μM) were added at the times indicated by the down-pointing and up-pointing arrows, respectively. Two traces are shown for each sensor. (**B**) *In vitro* studies of the periplasmic domain of BaeS (BaeS^P^). Isothermal titration calorimetry (ITC) binding curves describing the titration of CuSO_4_ into solutions containing the native BaeS^P^ (black) or mutant BaeS^P-H4/A^ (gray) domains, as well as titration of ZnCl_2_ into a solution of BaeS^P^ (light gray). The baseline-corrected raw data (upper panel) and the integrated curves (lower panel) are shown for each titration reaction series.

The periplasmic domain of BaeS consists of 130 amino acids, four of which are His (at positions 45, 65, 87, and 101). Using site-directed mutagenesis, we replaced these His residues with Ala, yielding the mutant BaeS^H4/A^. The mYFP-tagged BaeS^H4/A^ mutant protein maintained its association with the cell membrane (S3 Fig). Fig 5A shows the response of this mutant (open symbols) to copper compared with the response of the native sensor (filled symbols). The BaeS^H4/A^ mutant exhibited a substantially reduced response, indicating that the BaeS periplasmic domain plays a major role in the response to copper.

To test whether copper ions interact directly with the periplasmic domain of BaeS, we cloned and purified monomers of the BaeS periplasmic domain (BaeS^P^; 15kD) using a cleavable N-terminal His-tag (S4A Fig). In general, sensory kinases are composed of alpha-helical regions connected by linkers with varying degrees of flexibility. The CD spectrum of the BaeS^P^ protein exhibited minima at approximately 208 nm and 220 nm (S4B Fig), indicating that this protein indeed folded into alpha-helical structures together with disordered or flexible elements. To test whether Cu^2+^ ions interacts directly with BaeS^p^, we performed isothermal titration calorimetry (ITC). As shown in Fig 5B, Cu^2+^ exhibited a strong tendency to bind to BaeS^P^ (black line and symbols) with approximately 1:1 stoichiometry. The binding curve could not be interpreted directly because the measured changes in enthalpy originated from both the copper-binding event and the clustering event. However, the changes in heat arising from the associations between the BaeS^P^ domains appear to be minimal. No binding was detected when the experiment was repeated with the mutant domain carrying the H4/A replacements (gray line and symbols). This BaeS^P-H4/A^ showed the same CD spectrum as BaeS^P^ (S4 Fig). In addition, Zn^2+^ displayed a much weaker affinity to the BaeS^P^ domain (light gray line and symbols). These data confirm that copper ions can directly interact with the periplasmic domain of BaeS.

### BaeS-dependent activation of the *spy* promoter

**Fig 6:**
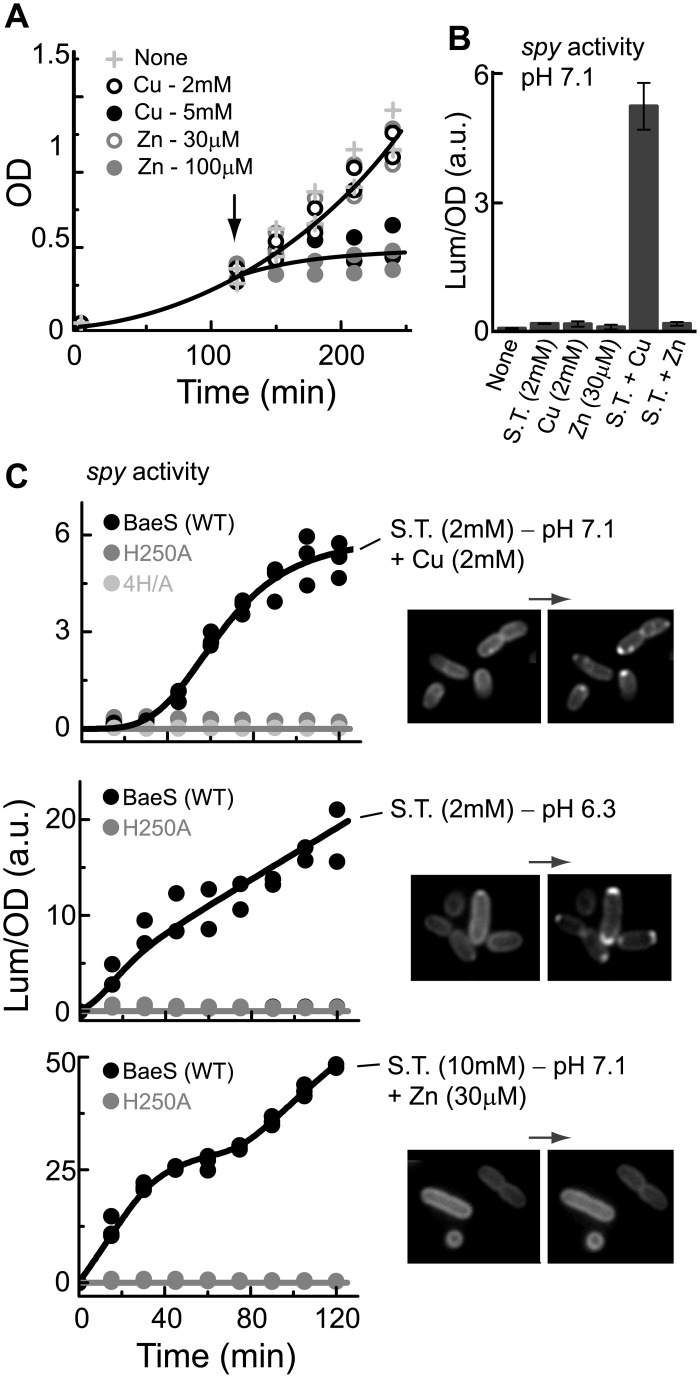
BaeS clustering is triggered by external conditions that also trigger BaeS-dependent *spy* promoter activation. (**A**) The effect of Cu^2+^ or Zn^2+^ on the growth of the MK1 strain (Δ*baeS*Δ*cpxA*). Bacterial growth was monitored by measuring the optical density (OD_600_) of the culture; CuSO_4_ or ZnCl_2_ were added at the times indicated by the arrows. (**B**) The luminescence (normalized to the OD_600_) measured from the MK1 cells supplemented with the *lux/spy* system and the *baeS*-*mYFP/baeR* construct 120 minutes after addition of 2 mM sodium tungstate, 2 mM CuSO_4_ or 30 μM ZnCl_2_ in different combinations and at different pH values, as labeled. (**C**) Time traces of the luminescence measured from the MK1 cells supplemented with the *lux/spy* system and either the *baeS*-*mYFP/baeR* (black symbols), *baeS*^*H250A*^-*mYFP/baeR* (gray symbols) or *baeS*^*4H*^*-mYFP/baeR* (light gray symbols) construct. Measurements were obtained after shifting the cells, at time zero, from the TB/pH 7 environment to the modified conditions, as labeled. Images of cells carrying the *baeS*-*mYFP/baeR* construct in either the TB/pH 7 environment (left) or after transfer to the indicated conditions (right) are shown.

As shown in Fig 1 (see also S1 Fig), the tagged BaeS sensor could trigger a response of the *acrD* promoter to indole and the *spy* promoter to sodium tungstate. The H250A substitution in the phosphorylation site of BaeS completely abolished the response of the *spy* promoter but only partially reduced the response of the *acrD* promoter. Thus, to study the potential effect of BaeS clustering on signaling, we focused on the *spy* promoter. Because the CpxAR and BaeSR systems were generally implicated in collaborative signaling [8, 37], we also constructed a new strain (MK1) in which both the *baeS* and *cpxA* genes were deleted. Finally, aiming for specific responses, we only imposed mild stresses that did not have a significant effect on bacterial growth, and performed all the experiments in TB medium with controlled pH. As shown in Fig 6A, the growth of the MK1 cells was considerably inhibited by 5 mM copper or 100 μM zinc, whereas 2 mM copper or 30 μM zinc did not significantly affect the bacterial growth; thus, the latter concentrations were used in subsequent experiments.

Under the conditions described above (TB, pH 7.1), no transcriptional response of the *spy* promoter could be observed with sodium tungstate, copper, or zinc (Fig 6B). In contrast, as shown in Fig 6B and 6C (black symbols), the *spy* promoter was activated by the following: (i) 2 mM sodium tungstate combined with 2 mM copper, at pH 7.1; (ii) 2 mM sodium tungstate only, at pH 6.3; or (iii) a higher concentration of sodium tungstate (10 mM) combined with 30 μM zinc, at pH 7.1. A lower sodium tungstate concentration (2 mM) combined with 30 μM zinc or sodium tungstate by itself (2 or 10 mM), all at pH 7.1, had no effect on transcription. All of the transcriptional responses observed here could be eliminated by the H250A mutation at the BaeS phosphorylation site (Fig 6C, gray symbols). We then looked at the formation of BaeS-mYFP clusters under the same conditions that activated the *spy* promoter. At the lower sodium tungstate concentration (2 mM), *spy* could be activated either by lowering the pH or by adding copper at pH 7.1 (conditions i and ii), and in both cases, BaeS clusters were clearly visible (Fig 6C). In contrast, no clustering could be detected in the presence of 10 mM sodium tungstate and 30 μM Zn^2+^ at pH 7.1 (condition iii).

## Discussion

Although several sensory kinases have been reported to form clusters [21–23], dynamic clustering of such sensors, triggered by specific environmental cues, has not been demonstrated in living cells. We now demonstrate that, in the presence of copper ions, the BaeS sensory kinase rapidly forms clusters (Figs 2 and 3), which then rapidly dissociate upon the addition of a divalent metal chelator (EDTA). The response of the BaeS sensors to copper was selective, as we did not observe a strong BaeS response to other divalent metals or strong responses to copper by other sensory kinases, although CusS exhibited a weak response to copper and BaeS exhibited a weak response to Zn^2+^ (Fig 4A and S2 Fig). These finding indicate that clustering of sensory kinases can be dynamic and stimulus dependent.

Clustering was observed even at the lowest expression level of BaeS tested, corresponding to a few hundred sensors per cell. In addition, BaeS clustering was reversible and was not apparent in the absence of copper or after addition of EDTA, and thus it is clearly not a direct effect of protein overexpression. The observed clustering of BaeS is unlikely to be triggered by the cytoplasmic mYFP tag, because His-to-Ala replacements in the periplasmic domain of the BaeS sensor substantially decreased the response to copper (Fig 5A). Furthermore, the EYFP-A206K tag used here has an immeasurably low tendency for self-association [24]. Finally, no response was observed with other mYFP-tagged sensors (Fig 4B). We therefore conclude that the observed response to copper is an inherent property of the BaeS sensor.

Several lines of evidence indicate that the periplasmic sensor domain of BaeS mediates the observed response to copper. Replacement of His residues with Ala in the periplasmic domain of BaeS substantially decreased the response to copper (Fig 5A). The four His replacements did not affect the structure of periplasmic domain of BaeS as shown by CD measurements (S4 Fig). Copper-dependent aggregation has also been reported for amyloid and prion proteins [35, 38], and in those cases, aggregation also depends on interactions between copper ions and His residues. In the case of BaeS, we demonstrate a direct interaction of copper with BaeS^P^ using ITC, which was abolished by four His-to-Ala replacements (Fig 5B). Other sensory kinases did not exhibit strong clustering response to copper (Fig 4B), suggesting that the response of BaeS to copper does not involve a generally conserved structure in the cytoplasmic domain of multiple sensor kinases. Finally, we found that the rapid response of BaeS to copper (Fig 3A) was consistent with the dynamics of the copper-induced quenching of periplasmic mCherry fluorescence (Fig 3B) and that EDTA can rapidly recover the anisotropy level of BaeS-mYFP after exposure to Cu^2+^ (Figs 2 and 3).

Figs 2 through 5 illustrate the ability of Cu^2+^ ions to interact with the BaeS periplasmic domain to promote the clustering of BaeS in cells resuspended in a defined minimal motility medium [27]. BaeS clustering also occurred in a richer growth-medium (TB) under conditions that promote BaeS-dependent activation of the *spy* promoter (Fig 6). In TB, an elevated level of Cu^2+^ ions or other metal ions were required for triggering both the clustering and transcriptional responses, possibly due to a chelating capacity of this medium [27]. However, the level of Cu^2+^ (2 mM), sodium tungstate (2 mM) or Zn^2+^ (30 μM) required to evoke responses still allowed normal bacterial growth. At pH 7.1, sodium tungstate, by itself, could not activate the *spy* promoter; however, when copper was also added or when the pH was lowered to 6.3, a clear activation of the *spy* promoter was observed. Both stimuli also triggered clustering of the BaeS sensor (Fig 6C). The pH effect could be due to structural changes in the protein caused by the protonation state of the His residues. Given that the pH of an *E*. *coli* culture is naturally lowered if cells are grown in a rich LB medium, these data are consistent with the observation that sodium tungstate [9] and copper [11] are stimulating effectors for BaeS in cells grown in LB.

The BaeS transcriptional responses are generally integrative and involve multiple two-component sensory systems [8, 37]. The fact that Zn^2+^ triggered transcriptional responses but did not trigger clustering of the BaeS sensors indicates that BaeS signaling can occur in the absence of clustering. However, other conditions that induced BaeS-dependent activation of the *spy* promoter also triggered BaeS clustering. Thus, the clustering of BaeS may contribute in an as yet undefined manner to the signaling of the BaeSR two-component system.

## Materials and Methods

### Strains and plasmids

The strains and plasmids used in this study are listed in S1 Table. All the strains are derivatives of the *E*. *coli* K-12 strain. A Δ*baeS* strain (JW2063C) was constructed from JW2063 (Δ*baeS*, Keio collection) by excision of the kanamycin cassette according to the procedure of Datsenko and Wanner [39]. BaeS was tagged with monomeric yellow fluorescent protein EYFP^A206K^ (mYFP) at its C-terminal cytoplasmic end via a short flexible linker (Gly Ser Gly Gly Gly). The histidine-to-alanine replacements in the periplasmic region of the BaeS protein were performed using the QuikChange Lightning Site-Directed Mutagenesis Kit (Agilent Technologies). The MK1 strain (*baeS*, *cpxA*) was prepared using P1 transduction from JW3882 (*cpxA)* into JW2063C.

### Growth conditions and induction levels

The cells were grown overnight in 1 ml of tryptone broth (TB; Bacto Tryptone 1% w/v, NaCl 0.5% w/v) diluted 100-fold in 10 ml of TB supplemented with the appropriate antibiotics and inducers. The cells were allowed to grow to an optical density at 600 nm (OD_600_) of approximately 0.4–0.5, after which they were washed and resuspended in motility buffer (10 mM potassium phosphate, 1 μM methionine, and 10 mM lactic acid; pH 7). The experiments were performed at room temperature (22–23°C).

### Fluorescence polarization measurements

The cells were immobilized on a coverslip and placed in a gold-plated flow chamber. The flow chamber was mounted on a Nikon FN1 microscope equipped with a 40× Plan Fluor objective (0.75 NA) and a 150 W xenon lamp (Hamamatsu, Bridgewater, NJ). The yellow fluorescent protein (YFP) was excited with linearly polarized light using a linear glass polarizer (Edmund Optics, Barrington, NJ), an ET508/6x excitation filter (Chroma Technology, Brattleboro, VT), and an FF520Di01 dichroic mirror. The fluorescence was collected using a FF01-542/27 emission filters (Semrock, Rochester, NY) and was split using a polarizing beam splitter cube (Newport, Irvine, CA). The parallel (*I*_*par*_) and perpendicular (*I*_*per*_) polarizations were monitored with photon counters (H7422P, Hamamatsu). The steady-state polarization of the emitted fluorescence is represented here by the fluorescence anisotropy, *r*, defined as (*I*_*par*_ − *I*_*per*_)/(*I*_*par*_+2*I*_*per*_), where *I*_*per*_ was corrected for imperfections of the optical system. The validation of the absolute fluorescence anisotropy was performed by adjusting the anisotropy recorded from an aqueous solution of fluorescein to zero. This calibration yielded an anisotropy level of 0.32 for purified YFP.

### Time-lapse fluorescence imaging

Cells expressing YFP-tagged BaeS receptors were immobilized on a coverslip and placed in a flow chamber. Fluorescence images were obtained using a Nikon Ti inverted microscope equipped with a 100× Plan Fluor objective (1.3 NA), a xenon lamp (Sutter Instruments), a camera (Andor Technology), and a ‘perfect focus’ feedback system to ensure constant focus between successive images.

### Quantification of expression levels of tagged sensors

The expression levels of the tagged sensors were estimated using previously described methods [23]. Except for in the experiments presented in Fig 2B (lower panel), the expression levels of the monomeric YFP (mYFP)-tagged sensors were estimated to be approximately 1,000 sensors per cell. In the experiments depicted in the lower panel of Fig 2B, the expression level in individual cells was estimated to be approximately 100–200 sensors per cell. The expression levels in single cells were estimated by comparing the total fluorescence emitted by the cell to that emitted by a single locus of the 240xTetO DNA operator-labeled YFP-tagged TetR DNA-binding protein.

### Purification of the BaeS periplasmic domain

The periplasmic region of the BaeS sensor (residues 38–168) or its mutant, BaeS^H4/A^, were cloned into an N-terminal His plasmid (pHis-1) under the control of the T7 promoter, which was transformed into bacterial strain C41(DE3). The bacteria were grown at 37°C, and when an OD_600_ of 0.6 was reached, IPTG (0.1 mM) was added. After 3 hours of induction, the cells were harvested, sedimented to form pellets, and maintained at -80°C. Next, the pellet from 0.6 L of bacteria was resuspended in 25 mM Tris HCl buffer (0.5 mM NaCl and 10% glycerol; pH 8) and 10 mM imidazole. The cells were lysed using a microfluidizer, and the soluble fraction was purified using a 3 ml nickel sepharose column and eluted using an imidazole gradient. The eluted 15 kD protein (eluted at 250 mM imidazole) was incubated with TEV protease at a ratio of 1:20 during overnight dialysis at 4°C. The cleaved protein was purified using the 3 ml nickel sepharose column. BaeS^P^ was eluted at the unbound peak and was further purified using a 200 ml Superdex 75 size exclusion chromatography column and 20 mM HEPES elution buffer (0.3 M NaCl and 5% glycerol; pH 7). This buffer was also used as the storage buffer. The protein purity was confirmed by Coomassie staining of an SDS-PAGE gel.

### Circular dichroism (CD) measurements

BaeS^P^ (15 μM) was dissolved in 20 mM HEPES buffer (100 mM NaCl and 2% glycerol; pH 7), and the protein concentration was measured using an ultraviolet (UV) spectrophotometer (UV-1650PC, Shimadzu, Japan). The CD spectra of the protein solution were recorded at 4°C in a 0.1 cm quartz cuvette using a J-810 spectropolarimeter (Jasco) in the spectral range of 195–260 nm.

### Isothermal titration calorimetry (ITC) measurements

ITC measurements were performed at 10°C using a calorimeter (ITC-200, MicroCal). BaeS^P^ (50 μM) was dissolved in 20 mM HEPES buffer (300 mM NaCl and 5% glycerol; pH 7). Next, CuSO_4_ or ZnCl_2_ (0.8 mM) was dissolved in the same HEPES buffer and titrated into the protein in the sample cell. As a control, metal solutions were titrated into a buffer solution, and the buffer was titrated into a protein solution. The metal solutions (2 μl) were injected into the protein sample in 4 s titration with a 200 s delay between injections to allow for equilibration [40]. The ITC data were analyzed using Origin 7.0 software.

### Luminescence assay

Bacterial strains JW2063C (Δ*baeS*) or MK1 (Δ*baeS* Δ*cpxA*) were transformed with a pBR2TTS plasmid [41] carrying the *Lux* protein under the control of the *acrD* (-274 up to +11) or the *spy* (-558 up to +88) promoter. For the functionality experiments of the mYFP-tagged BaeS, the JW2063C strain was also transformed with a pBAD33 plasmid carrying *baeS-mYFP/baeR* or the same plasmid with a histidine to alanine replacement of the histidine kinase residue (*baeS*^*H250A*^*-myfp/baeR*). For the controls, we used JW2063C cells transformed with an empty pBAD33 plasmid and JW2064 cells (Δ*baeR*) transformed with mYFP-tagged BaeS. All plasmids were under the control of an arabinose promoter (CamR). To measure the copper effect on BaeS signaling, we used three constructs: *baeS-mYFP/baeR*, *baeS*^*H250A*^*-myfp/baeR*, and *baeS*^*H4/A*^-*myfp*/*baeR*. Cells were grown overnight at 37°C, diluted 1:100 into LB or TB and grown until reaching an OD_600_ of 0.6. Afterward, the cells were exposed to various induction conditions. To measure the *acrD* promoter activity, we used 2 mM Indole, and to measure the *spy* promoter activity, we used 2 mM or 10 mM sodium tungstate, 2 mM copper, 30 μM zinc and combinations of sodium tungstate and copper or zinc, each at various pH levels. Then, 100 μl of the cells was transferred to a 96-well plate for measurements of both the kinetic luminescence during 4 hours of growth and the OD of the samples at 600 nm. The experiment was repeated three times using a plate reader (Synergy 2, BioTek). The results are expressed as the arbitrary units of luminescence divided by the OD at each time point.

## Supporting Information

S1 Fig The BaeS-mYFP-dependent response of the *acrD* promoter to indole. (PDF)

S2 Fig Copper-induced clustering of the bacterial sensory-kinase CusS. (PDF)

S3 Fig Fluorescence images of BaeS^4H/A^ -mYFP. (PDF)

S4 Fig Analytical gel filtration and circular dichroism spectrum of BaeS^P^. (PDF)

S1 Table List of plasmids and strains. (PDF)

## Abbreviations

mYFP: monomeric yellow fluorescent protein
FRET: fluorescence resonance energy transfer
IPTG: isopropyl β-D-thiogalactopyranoside
CD: circular dichroism
ITC: isothermal titration calorimetry
