# Supplementary material for: Dynamic Clustering of the Bacterial Sensory Kinase BaeS
Source: PLoS One. 2016 Mar 7;11(3):e0150349. doi: 10.1371/journal.pone.0150349 (PMC4780735; doi:10.1371/journal.pone.0150349)
Supplement: S1 Fig — (PDF) [file pone.0150349.s001.pdf]

Supporting information

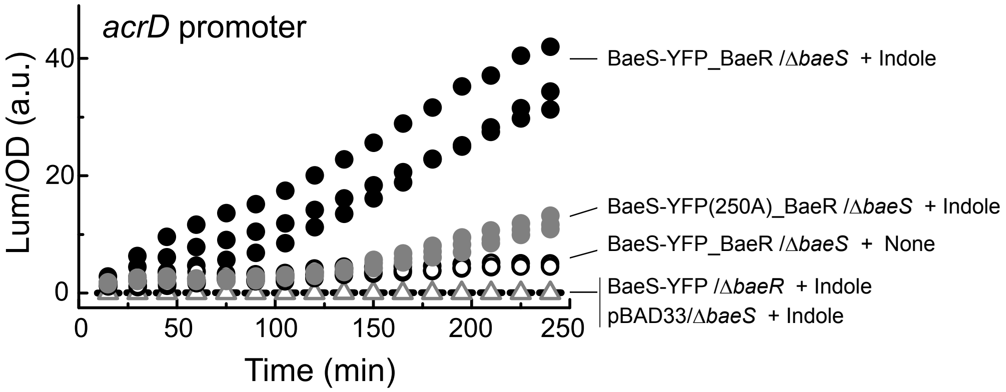

Fig. S1 Time traces of the luminescence, normalized to the optical density (OD) of the culture, measured from Δ*baeS* cells (JW2063) carrying the *lux* system under the control of the *acrD* promoter and after the addition, at t=0, indole (2 mM). Cells were supplemented also with either empty pBAD33 plasmid, a plasmid carrying the tagged sensors *baeS-mYFP*, a plasmid carrying the mutant sensor *baeS*^{H250A}*-mYFP*, or a plasmid carrying the untagged *baeS* sensor. In all cases, the *baeR* response regulator gene was also cloned in tandem. Measurements were done in LB medium. Each experiment was repeated three times.

Figure S1
