# Supplementary material for: Dynamic Clustering of the Bacterial Sensory Kinase BaeS
Source: PLoS One. 2016 Mar 7;11(3):e0150349. doi: 10.1371/journal.pone.0150349 (PMC4780735; doi:10.1371/journal.pone.0150349)
Supplement: S2 Fig — (PDF) [file pone.0150349.s002.pdf]

Supporting information

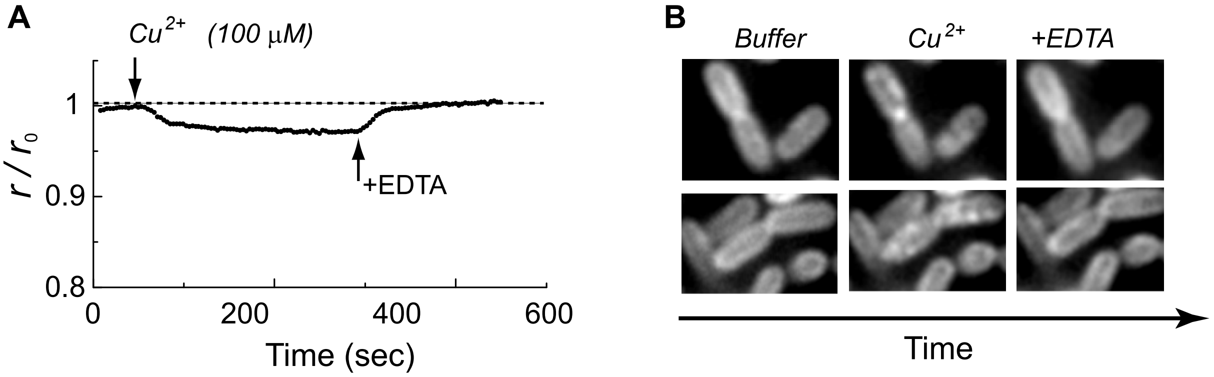

Fig. S2 The response of the bacterial CusS Sensorykinase to copper. (**A**) Fluorescence anisotropy trace measured from Δ*cusS* cells expressing mYFP-tagged CusS. (**B**) Time-lapse images of similar cells as in (A) mounted in a flow chamber and imaged successively in motility buffer, 100 μM Cu^{2+}, and 100 μM EDTA.

Figure S2
