# Supplementary material for: Dynamic Clustering of the Bacterial Sensory Kinase BaeS
Source: PLoS One. 2016 Mar 7;11(3):e0150349. doi: 10.1371/journal.pone.0150349 (PMC4780735; doi:10.1371/journal.pone.0150349)
Supplement: S3 Fig — (PDF) [file pone.0150349.s003.pdf]

Supporting information

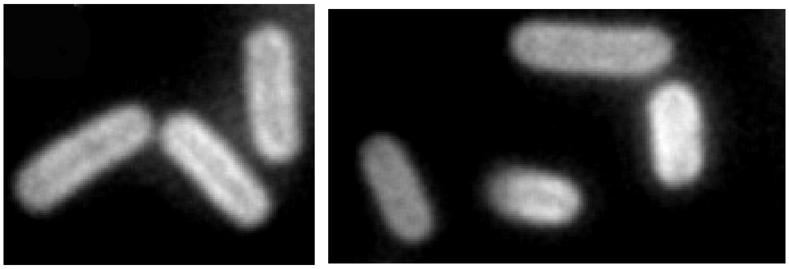

Fig. S3 The BaeS^{4HA} mutant receptor is still bound to the membrane. Fluorescence images of mYFP-tagged BaeS^{4HA} mutant receptor in wild type cells. No induction was used.

Figure S3
