# Supplementary material for: Dynamic Clustering of the Bacterial Sensory Kinase BaeS
Source: PLoS One. 2016 Mar 7;11(3):e0150349. doi: 10.1371/journal.pone.0150349 (PMC4780735; doi:10.1371/journal.pone.0150349)
Supplement: S4 Fig — (PDF) [file pone.0150349.s004.pdf]

Supporting information

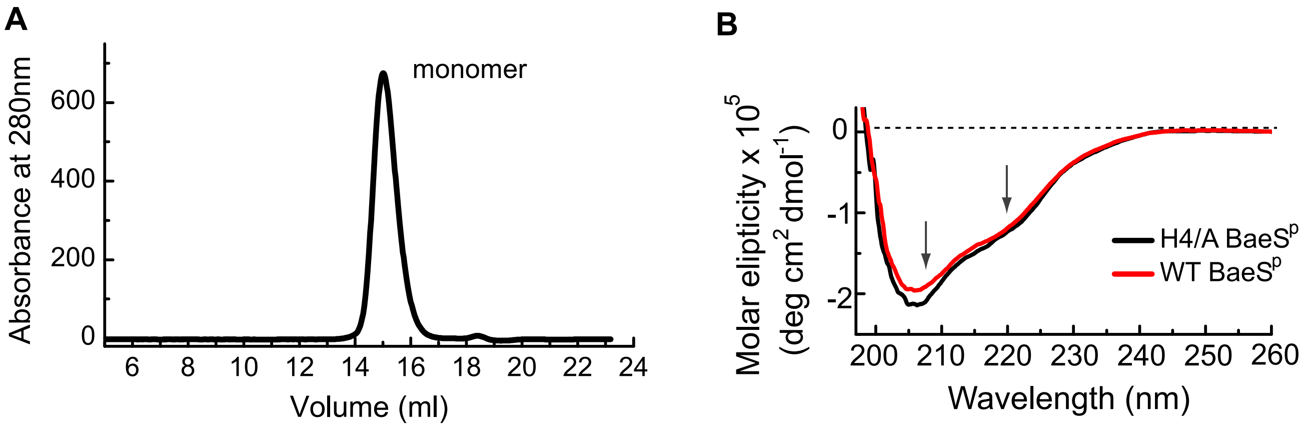

Fig. S4 Analysis of BaeS^P. (**A**) Analytical gel filtration of BaeS^P, shows that the protein elutes as a monomer of size 15Da. (**B**) The circular dichroism spectrum of BaeS^p (15 µM) measured at 4°C. Minima occurred at approximately 208 nm and 220 nm (arrows), as is typical for α-helical structures.

Figure S4
