# Supplementary material for: Dynamic Clustering of the Bacterial Sensory Kinase BaeS
Source: PLoS One. 2016 Mar 7;11(3):e0150349. doi: 10.1371/journal.pone.0150349 (PMC4780735; doi:10.1371/journal.pone.0150349)
Supplement: S1 Table — (PDF) [file pone.0150349.s005.pdf]

Table S1- List of plasmids and strains

Plasmids	Description	Vector	Source
	EYFP		Clontech
	mYFP		Ref. (*1*)
pES20	*baeS-mYFP*	pTrc99A	Ref. (*2*)
pAVM103	*mYFP-mYFP*	pTrc99A	This study
pAVM146	*baeS-mYFP*	pBAD33	This study
pAVM35	*tetR-mYFP*	pBAD33	Ref . (*2*)
pAVM151	*Free mCherry*	pBAD33	This study
pAVM170	*torT-mCherry*	pBAD33	Ref . (*2*)
pES-27	*envZ-mYFP*	pTrc99A	Ref . (*2*)
pAV127	$phoQ^{N202R}$ *-YFP*	pTrc99A	This study
pAE-1	*cusS-mYFP*	pTrc99A	Ref . (*2*)
pES-28	*cpxA-mYFP*	pTrc99A	Ref . (*2*)
pAVM244	*baeS(H45A,H65A,H87A,H101A)-mYFP*	pTrc99A	This study
pAVM213	*baeS(107-505)*	pHis-1	This study
pAVM252	*baeS(107-505) (H45A,H65A,H87A,H101A)*	pHis-1	This study
pAVM266	*baeS(H250A)-mYFP-BaeR*	pBAD33	This study
pAVM267	*baeS-mYFP-BaeR*	pBAD33	This study
pAVM271	*baeS-baeR*	pBAD33	This study
pAVM293	*baeS(H45A,H65A,H87A,H101A)-mYFP-baeR*	pBAD33	This Study
pAVM267	*baeS-mYFP-baeR*	pBAD33	This study
pAVM224	*P-acrD-lux*	pBR2TTS	This study
pAVM225	*P-spy-lux*	pBR2TTS	This study

Strains	Description	Source
JW 2063C	ΔBaeS strain	Keio collection
JW2064C	ΔBaeR strain	Keio collection
IL-2	TetO cassette strain	Ref. (*3*)
MK1	ΔBaeS, CpxA:Kan	P1 from JW3882 into JW2063C
C41(DE3)	BL21 derivate	Lucigen

1. D. A. Zacharias, J. D. Violin, A. C. Newton, R. Y. Tsien, *Science* 296, 913 (2002).
2. E. Sommer, M. Koler, V. Frank, V. Sourjik, A. Vaknin, *PLoS ONE* 8, e77708 (2013).
3. I. F. Lau *et al.*, *Mol. Microbiol.* 49, 731 (2003).
